# ‘I felt like a human being’—An exploratory, multi‐method study of refugee involvement in the development of mental health intervention research

**DOI:** 10.1111/hex.12990

**Published:** 2019-11-09

**Authors:** Georgina Warner, Zaruhi Baghdasaryan, Fatumo Osman, Elin Lampa, Anna Sarkadi

**Affiliations:** ^1^ Department of Public Health and Caring Sciences Uppsala University Uppsala Sweden; ^2^ School of Education, Health and Social Studies Dalarna University Falun Sweden

**Keywords:** group dynamics, mental health, observation, patient and public involvement, refugees

## Abstract

**Background:**

Great advancements have been made in patient and public involvement (PPI), including the development of guidance on how to conduct, report and evaluate PPI. Despite these efforts, the evidence base remains relatively weak. A substantive methodological development is required. This is particularly important for vulnerable groups within society, for whom PPI can be challenging but has the potential to play a transformative role in shaping research.

**Objectives:**

To describe the group dynamic characteristics and immediate impact of PPI from the user representatives’ perspective in a case study of refugee involvement in the development of mental health intervention research. To pilot and methodologically appraise the Active Involvement of Users in Research Observation Schedule and Questionnaire.

**Design:**

The Active Involvement of Users in Research Observation Schedule and Questionnaire were administered together with a focus group discussion.

**Setting:**

‘Refugee Advisors’ were involved in the development of a randomized controlled trial protocol evaluating a brief group intervention for refugee children experiencing symptoms of post‐traumatic stress in Sweden.

**Results:**

The multi‐method approach demonstrated good feasibility. There were clear examples of how the advisors influenced research development. The advisors described a perceived impact on the research, equality and acceptance, and knowledge gain. A sense of appreciation and empowerment was also interpreted. However, potential issues relating to the relevance of contributions and use of an interpreter were identified.

**Discussion and conclusion:**

The methodological approach piloted in this study offers a promising, rigorous way to evaluate PPI. The research tools require further refinement and validation.

## 1 INTRODUCTION

Patient and public involvement (PPI) refers to an active partnership between patients and/or members of the public and researchers.^1^ Involvement is distinct from participation in research: patients and/or the public contribute to the research process as advisors, and possibly as co‐researchers.^1^ Involving ‘vulnerable’ groups within society, such as refugees, in research development can be challenging, but has the potential to play a transformative role in shaping research. Workshops with various stakeholders including PPI representatives can be an effective way to establish priority research areas that are meaningful to patients, clinicians and researchers alike.^2^ Patient representatives are able to make judgements based on their understanding of their condition and may have aspirations and thoughts about health outcomes that mental health‐care professionals and researchers may not have considered. PPI can support motions to overcome stigma, including accurate, inclusive communications about conditions and the intentions of research projects. It can help to inform sensitive and efficient recruitment strategies and, importantly, support truly informed consent. This can be particularly complex given the various languages potential refugee participants may use. It is common for researchers to exclude certain populations on linguistic grounds, a practice which may reflect on research outcomes and in turn may affect the provision of services to linguistically diverse populations.^3^ Language, culture, religion, social norms and experiences of oppression may also make it difficult to obtain accurate responses to research questions.^4^ The format of the research is another important consideration. Interviews may raise suspicion, given the long interviews as part of the asylum process, and focus groups could stimulate tension if participants come from different backgrounds.^4^

### 1.1 Standardization and evaluation of PPI

Thorough guidance on how to conduct and report PPI, such as INVOLVE (see invo.org.uk) and the Guidance for Reporting Involvement of Patients and the Public checklists (GRIPP^5^ & GRIPP2^6^), has been developed in an effort to standardize the process. Moreover, PPI evaluation frameworks have been developed to guide PPI impact assessment, such as the Public Involvement Impact Assessment Framework (PiiAF; see piiaf.org.uk). There is clear support for the importance of evaluating the impact of PPI^7^ and reviews of the literature indicate a growing research interest.^8^, ^9^

Yet, despite these efforts, the evidence base remains relatively weak. An international effort is required to improve the PPI evidence base, as the majority of evaluative literature comes from the UK.^8^, ^9^ Substantive methodological development is needed, including methods for assessing impacts both qualitatively and quantitatively. Evaluative data on PPI are often brief, narrative descriptions, which reflects the lack of robust tools specifically developed to assess PPI.^8^ Attempts to quantitatively assess the impact of PPI have been carried out, but have been critiqued for the lack of sufficient attention to (a) the context in which involvement takes place and (b) the way it is carried out.^10^ Yet, a fine balance is required as scientific approaches designed around a specific context are difficult to extrapolate beyond that given context.^10^ Staley^10^ maintains the value of evaluating the impact of PPI but calls for more detailed accounts of PPI experiences; she argues that researchers’ accounts of their experience potentially provide a source of insight and learning to influence others.

There is a strong tendency to report on the beneficial influence PPI has on research and the research process, with few papers reporting on negative influence.^8^ Costs associated with PPI and co‐production could include adverse effects on the research itself, the research process, personal and professional risks for researchers and stakeholders and risks to the wider cause of scholarship.^8^, ^11^ The positive reports could indicate the benefits of PPI far outweigh the challenges, or it may indicate publication bias.^8^ It could be that methodological bias is also contributing.

Behavioural observation, the systematic recording of behaviour by an external observer, could bring great insight to the factors and agents that contribute to successful PPI. Yet, this appears to be an underexplored research area. A semi‐structured observational approach would allow for mechanistic elements of PPI to be coded against a set protocol and for the context‐specific details to be recorded in the form of observational notes, and thus also providing a detailed experiential account. This approach would strike the aforementioned balance between context and extrapolation called for by Staley.^10^ By utilizing an observational approach whereby the observer is not part of the social exchange being observed, the aspect of reciprocity (i.e. the practice of exchange for mutual benefit) can be observed. The structure of reciprocity in exchange affects the solidarity of bonds that arise from exchange.^12^ If PPI is to represent a partnership approach to developing and conducting research, then high levels of reciprocity are important. Incorporation of the theory of reciprocity into the evaluation of PPI can be further strengthened by a triangulation of data by which researchers and PPI representatives are given the opportunity to directly reflect on the PPI both in terms of the mechanisms and perceived impact, in addition to being observed.

### 1.2 Evaluation of PPI group dynamics

**Figure 1:**
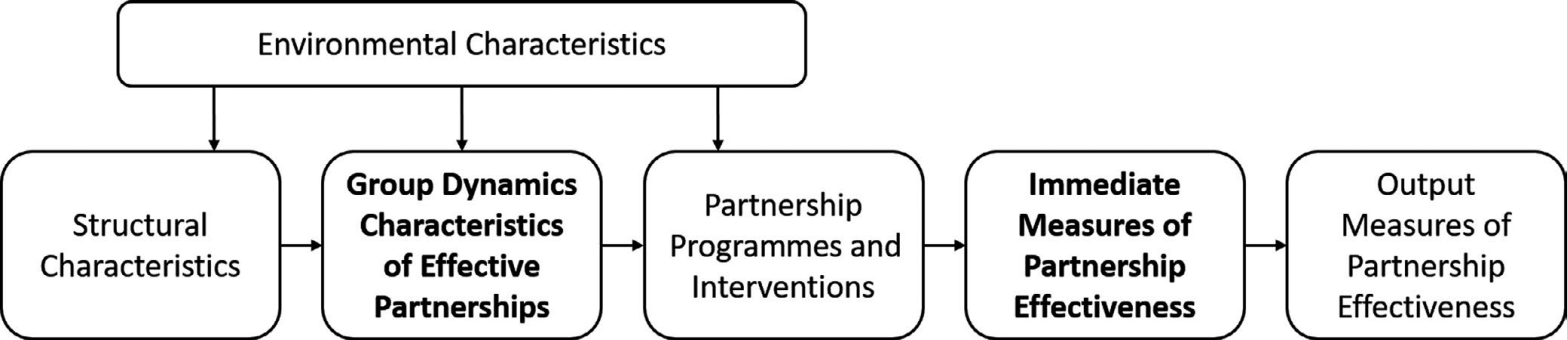
Overview of Schulz et al (2002) conceptual framework for assessing the effectiveness of participatory research

Group dynamics are the interpersonal processes that determine how group members relate to and engage with each other and what the group achieves.^13^ There are several influential aspects to consider when studying group dynamics, including communication processes and interaction patterns; interpersonal attraction and cohesion; social integration and influence; and power and control.^13^, ^14^ Schulz et al^15^ developed a conceptual framework for assessing the effectiveness of participatory research, in which group dynamic characteristics play a central role (Figure 1). Derived from group process literature, the framework acknowledges the reliance on group effectiveness in achieving short‐term and long‐term participatory research goals. It is proposed that, in order to progress to research outputs that are both influenced by and meaningful to user representatives and researchers alike, the process of meeting and discussing the research must facilitate meaningful involvement. Collaboration is dual‐faceted in that both the content of the problem to be solved and the relational aspects of the group need to be managed; and more successful groups are more responsive to one another.^16^, ^17^ Schulz et al^15^ developed a questionnaire that explored group dynamic aspects of participatory research; however, to the best of our knowledge, no observation protocol examining group dynamics in PPI exists.

## 2 ACTIVE INVOLVEMENT OF USERS IN RESEARCH OBSERVATION SCHEDULE AND QUESTIONNAIRE

The Active Involvement of Users in Research Observation Schedule is a semi‐structured observation protocol developed to objectively assess aspects of group dynamics in the context of PPI research meetings. The observation protocol consists of twelve observable behaviours relating to the interpersonal relations between researchers and PPI advisors; the nature of advisor contributions; and how the advisors guide research development. Each category consists of positive and negative behaviours. See Table 1 for the full coding list and descriptions. There is an accompanying paper‐based assessment form that allows attendees to independently and anonymously grade the meeting on a Likert scale from 1 (Not at all) to 5 (A lot) on a list of items that correspond to those on the observation pro‐forma.

**Table 1:** Active Involvement of Users in Research Observation Schedule coding list and descriptions

Observation/Coding	Description	Group dynamic component
interpersonal relations		
1. Positive interactions	How the advisors and the research team interact. Take note of positive interactions (e.g. humorous or appreciative remarks).	Interpersonal attraction and cohesion
2. Reference to advisors’ expertise	How the researchers refer to the advisors and/or advisor input. Take note of comments that infer skill or knowledge (e.g. use of terms such as ‘expert’, ‘important’, ‘valuable’, ‘helpful’ ‘interested to know what you think’).	Social integration and influence
3. Linguistic barriers to advisor participation	The accessibility of the conversation to the advisors. Take note of any linguistic barriers (e.g. scientific language that is difficult to understand; interpretation issues, such as insufficient time to translate and miscommunications).	Communication processes and interaction patterns
4. Advisors showing a lack of interest/ being disengaged	Advisor body language. Take note of gestures or actions that infer a lack of interest (e.g. yawning, looking away from the point of focus, looking at mobile phone, doodling, checking the time).	Interpersonal attraction and cohesion
nature of advisor contributions		
5. Invitations to speak	Researchers directly asking advisors to comment. This can be a specific question, or asking for any further thoughts on a point of discussion.	Communication processes and interaction patterns
6. Taking the initiative to speak	Advisors providing comments without being directly asked. This can include an advisor spontaneously adding to a response of another advisor (even if the first advisor was directly asked a question).	Communication processes and interaction patterns
7. Passively agreeing with researchers	Advisors’ responses to researcher questions. Take note of occasions when advisors appear to agree with researchers without active consideration. It is the level of engagement that is important. Active agreement with researchers should not be scored negatively.	Power and control
8. Offering insights appearing irrelevant to discussions	Advisors making comments that do not appear to be connected to the current conversation, or providing an unnecessary level of detail.	Communication processes and interaction patterns
how advisors guided research development		
9. Challenging and suggesting alternatives to researchers	Advisors questioning the logic or approach of the researchers and/or providing different option(s) to consider. The relevance of challenges should be considered. Only constructive challenges should be scored positively.	Social integration and influence
10. Incorporation of advisors’ ideas in research planning	Advisor comments influencing the research plan. This could be in relation to any aspect of the research (e.g. questionnaire selection or modifications, study age range, recruitment strategies, interpretation of findings etc). It can include intentions to act on advisors’ comments (e.g. ‘We should try to pilot that questionnaire with more people’).	Social integration and influence
11. Ideas being ignored/treated with disregard	Active consideration of advisors’ ideas. Take note of occasions when advisors’ input appears to be overlooked. Actively challenging the advisors’ input should not be scored negatively. It is a lack of consideration that is the focus.	Power and control
12. Decisions made without the input of advisors	Research project decisions or intentions. Take note of when decisions appear to be made and whether advisors were involved in the process. Be mindful of decisions made in break‐out group formats or during breaks. The decision does not have to necessarily reflect the advisors’ choice, but their input should have been sought/offered and considered.	Power and control

The core components of group dynamics^13^, ^14^ were used to guide the construction of the evaluation instruments (Table 1). Previous findings from PPI evaluations were used to place the group dynamic components in context. For instance, the communication processes and interaction patterns attribute of ‘Linguistic barriers to advisor participation’ was derived from user representative reports of inaccessible research language,^18^ as well as the potential for multi‐lingual group meetings. ‘Reference to advisors’ expertise’, considered an attribute of social integration and influence, was included in the protocol due to reports of assumed lack of knowledge and associated frustration from user representatives.^19^, ^20^, ^21^, ^22^ ‘Ideas being ignored/treated with disregard’, considered an aspect of power and control, was included in light of descriptions of user representatives’ ideas not being listened to or marginalized within discussions.^18^, ^21^

The intention is for the evaluation instruments to be applied to various PPI contexts and, thus, emphasis is placed on group dynamic characteristics rather than environmental or structural characteristics. It is also envisioned that the instruments are used within a multi‐method research design, which includes insights from interviews and/or focus groups. This triangulation of data enables the group dynamics assessment to be placed in the context of immediate or output measures of partnership effectiveness in line with the conceptual framework developed by Schulz et al^15^ (Figure 1). The case study provided in this paper utilizes the Active Involvement of Users in Research Observation Schedule and Questionnaire together with a focus group method to explore both PPI group dynamic characteristics and the immediate impact of PPI from the user representatives’ perspective.

### 2.1 Objectives

The objectives of the present paper are to:

1. Describe (a) group dynamic characteristics and (b) immediate impact of PPI from the user representatives’ perspective in a case study of refugee involvement in the development of mental health intervention research.
2. Pilot and methodologically appraise the Active Involvement of Users in Research Observation Schedule and Questionnaire.

## 3 CASE STUDY: REFUGEE INVOLVEMENT IN THE DEVELOPMENT OF MENTAL HEALTH INTERVENTION RESEARCH

### 3.1 Setting

The Child Health and Parenting (CHAP) research group at Uppsala University in Sweden received funding to develop a randomized controlled trial (RCT) to evaluate a brief group programme for refugee children experiencing symptoms of post‐traumatic stress. The research group invited refugees to be involved in the trial protocol development, who were referred to as ‘Refugee Advisors’. Information leaflets advertising the Refugee Advisor positions were created in Swedish, English, Arabic, Somali and Tigrinya. Members of the research team (ZB & EL) visited local education centres, where immigrant families attend societal orientation courses, and presented the details of the project. Applications (n = 8) were received via an expression of interest form. Four Refugee Advisors (3 parents and 1 youth) were selected based on how close their personal situation matched that of the intended study participants (i.e. children aged 8 or above showing symptoms of post‐traumatic stress who have resided in Sweden for 5 years or less) and their description of their motivation for taking part. The number of advisors chosen was to achieve a balance across roles within the group, including the advisors, core research team and international project advisors. The Refugee Advisors attended a one‐day meeting at Uppsala University, where presentations were made on related research and group discussion on the trial design (e.g. recruitment strategy, outcome measures, participant timeline) took place. The meeting was conducted in English and an English‐to‐Arabic interpreter attended the meeting. The Refugee Advisors were compensated for their time at an hourly rate. Nine researchers who attended the meeting, including international project advisors from UK, Norway and United States, provided feedback on the PPI group dynamics as part of the present study.

### 3.2 Data collection

During the 8‐hour research meeting, group dynamic characteristics were observed using the Active Involvement of Users in Research Observation Schedule. Observations were carried out by a member of the research team (ZB), who was briefed on the data collection requirements and not involved in the research meeting activity to ensure the observation was not compromised. Observations were ‘light touch’ in order to minimize intrusion and encourage normal behaviour; however, Refugee Advisors and researchers were aware they were being observed. At the end of the observed session, researchers (n = 9) were asked to complete the Active Involvement of Users in Research Questionnaire anonymously and the Refugee Advisors were asked to take part in a focus group discussion. According to recommended focus group methodology,^23^ a semi‐structured question guide was developed by the research team, aiming to give insight on the immediate impact of PPI from the user representatives’ perspective, including the perceived impact of Refugee Advisors on the research and impact on the Refugee Advisors as individuals. The focus group lasted 35 minutes and was facilitated by a moderator (FO) and an assistant moderator (ZB), who took notes during the discussions and made sure the moderator did not overlook any participants trying to add comments. The interpreter was used in the focus group, but both moderators also have conversational language proficiency in Arabic, that is they are able to carry on a conversation in the language although not fluently. During the focus group, Arabic, English and Swedish languages were used. The research meeting and focus group discussions were audio‐recorded.

### 3.3 Data analysis

The observations were coded according to the pro‐forma by the second author (ZB), who carried out the live observation. The first author (GW) reviewed the observations and the appropriateness of the codes. The scores from the questionnaires were summed for all the researchers and divided by the number of researchers to provide a mean score for each item (Table 2). The focus group discussion was transcribed verbatim, translated into English and analysed using a thematic approach.^24^ The analysis categories were deduced from the question guide topics, but the conception of themes was data‐driven. The first author reviewed the transcript and derived a set of themes and subthemes from the discussion. The second author independently reviewed the transcript and the appropriateness of the themes/subthemes. The first author then coded the transcript by categorizing relevant statements under themes and subthemes.^25^ The second author reviewed the coded statements. Disagreements were resolved through discussion, with the audio recordings consulted if needed. The other authors verified the results of the analysis.

**Table 2:** Overview of observation protocol and questionnaire components, including observation levels and questionnaire mean scores

	Observation level (Frequency)	Mean score (Out of 5)
interpersonal relations		
Positive interactions (+)	High	4.2
Reference to advisors’ expertise (+)	Low/Moderate	4.1
Linguistic barriers to advisor participation (−)	High	3.4
Advisors showing a lack of interest or being disengaged (−)	Moderate	1.3
nature of advisor contributions		
Invitations to speak (+)	High	4.8
Taking the initiative to speak (+)	Moderate/High	2.3
Passively agreeing with researchers (−)	Low	1.7
Offering insights appearing irrelevant to discussions (−)	Low	2.8
how advisors guided research development		
Challenging and suggesting alternatives to researchers (+)	Moderate	2.9
Incorporation of advisors’ ideas in research planning (+)	Low	3.9
Ideas being ignored/treated with disregard (−)	Low	1.1
Decisions made without the input of advisors (−)	Low	1.9

### 3.4 Ethics

Ethical approval was granted for the study, as part of the associated RCT ethics submission, by the Regional Ethical Review Board in Uppsala (Ref. 2018/382) on 28th November 2018. The nature of the Refugee Advisors' involvement was discussed with them when recruited and again a few weeks prior to the research meeting (the setting for the study), which included the research intentions (i.e. the observation and the focus group discussion). There was no obligation to take part in the research; they could be an advisor without taking part in the research. The second author (ZB), who has conversational language proficiency in Arabic, was in regular contact with the advisors and available to answer any questions the advisors had in the lead up to the meeting. The decision was taken to record consent orally, rather than in writing. A key reason for this was that some advisors were illiterate. We were also conscious of the power dynamic relating to the consent procedure. A prepared statement was read aloud to the advisors, with assistance from an interpreter, and the advisors were asked to state whether or not they were happy to proceed. The statement included the following: the research intentions, including the nature of the questions they would be asked in the focus group; that we would write about the research findings; that no names would be used in reports or papers; and that the conversation would be audio‐recorded and safely stored so we could listen to it again.

## 4 RESULTS

**Figure 2:**
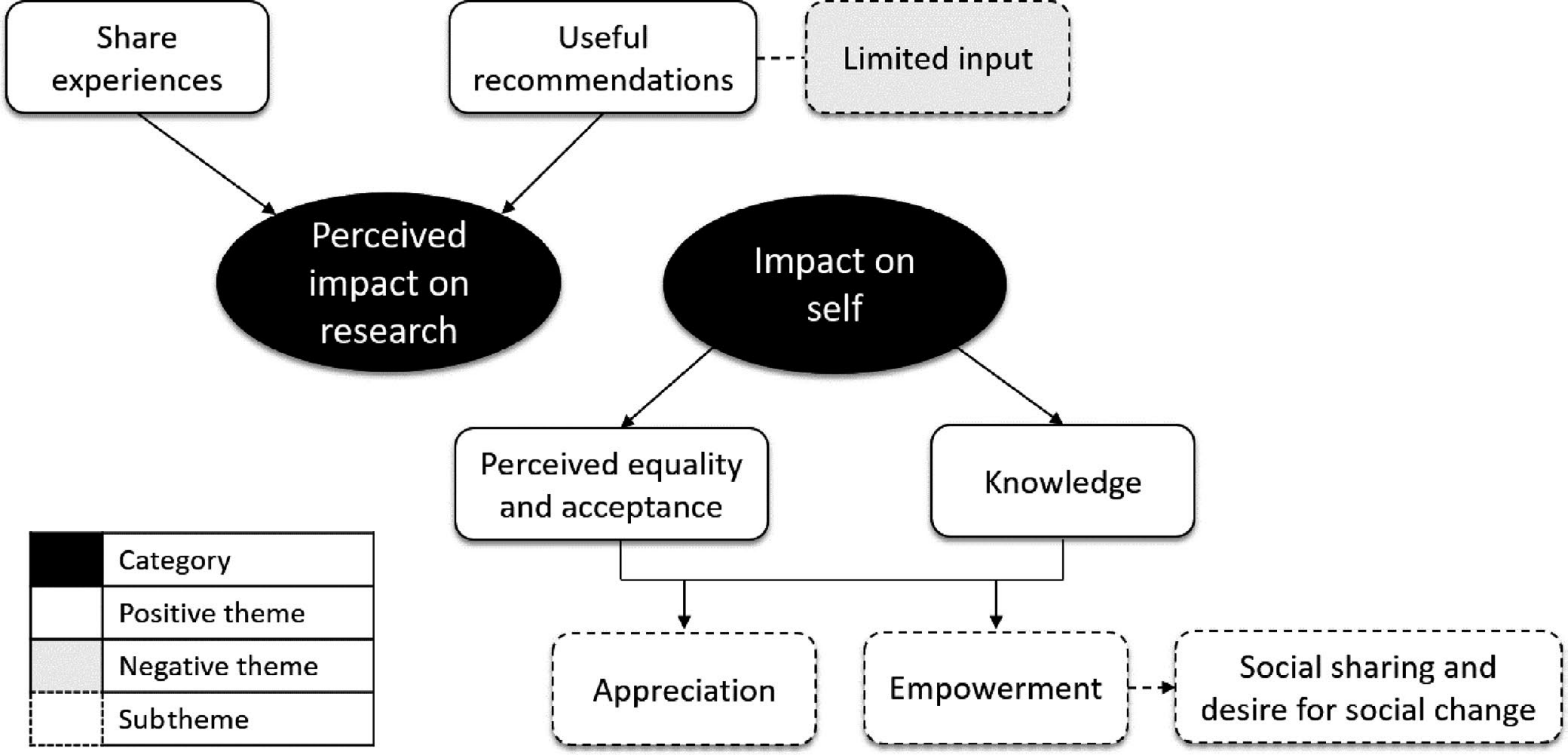
Refugee Advisor focus group themes

The results are presented with insights from the Active Involvement of Users in Research Observation Schedule and Questionnaire first, covering group dynamic characteristics (Table 2), followed by insights from the focus group, covering the immediate impact of PPI from the user representatives’ perspective (Figure 2).

### 4.1 Interpersonal relations

Positive interactions between the Refugee Advisors and the research group were frequently observed throughout the meeting. There were many humorous remarks made, resulting in group laughter. These were related to personal attributes of the attendees (names), the topic of the meeting (supporting refugee children) and the process (translations between languages). There were also a number of appreciative remarks (e.g. ‘I am deeply touched and grateful to be here. I feel wonderful, thank you’). The frequency of ‘positive interactions’ was also scored highly by researchers (mean score of 4.2 out of 5). Reference to the Refugee Advisors’ expertise (e.g. researchers stressing the importance of having equal voices in the discussion) was observed, but to a lesser extent. However, researchers scored ‘reference to advisors’ expertise’ relatively highly (mean score of 4.1 out of 5). ‘Linguistic barriers to participation’ were commonly observed, which included issues arising from interpretation. There were practical interpretation issues, such as the interpreter not having sufficient time to translate a video presentation. Miscommunications were also evident, including the difference between accompanied and unaccompanied refugee minors and accessing voluntary support opposed to volunteering. Broader interpretation issues included the interpreter neglecting to translate some speech that was not directed towards the advisors and the interpreter offering personal insights instead of those of the advisors. The frequency of ‘linguistic barriers to participation’ was also scored relatively highly by researchers (mean score of 3.4 out of 5). There were several observations of ‘advisors showing a lack of interest or being disengaged’; however, the researchers rated the frequency relatively low (mean score of 1.3 out of 5).

### 4.2 Nature of advisor contributions

Throughout the meeting, Refugee Advisors were invited to speak about various aspects of the study, including mental health help‐seeking behaviours in refugee youth; cultural sensitivities; participant recruitment strategies; and questionnaire item relevance and acceptability. ‘Invitations to speak’ frequency was rated very highly by researchers (mean score of 4.8 out of 5). Refugee Advisors taking the initiative to speak was also commonly observed, but was scored as relatively infrequent by researchers (mean score of 2.3 out of 5). ‘Passively agreeing with researchers’ and ‘offering insights appearing irrelevant to discussions’ were infrequently observed. However, ‘offering insights appearing irrelevant to discussions’ received a moderate frequency score from researchers (mean score of 2.8 out of 5).

### 4.3 How advisors guided research development

There was a moderate level of Refugee Advisors challenging and suggesting alternative ideas to researchers observed (e.g. recruitment strategy, study age range). This was reflected in the researchers’ scoring (mean score of 2.9 out of 5). There were few (but conceivably significant) observations of incorporating Refugee Advisors’ ideas into the research planning relating to: (a) the recruitment strategy; (b) addressing different backgrounds of refugee youth in introduction sessions; and (c) further testing of the validity of a suggested outcome measure. However, researchers scored the frequency of incorporating the ideas of the advisors into research planning relatively highly (mean score of 3.9 out of 5). ‘Ideas being ignored/treated with disregard’ and ‘decisions made without the input of advisors’ were infrequently observed. They were also rated low by researchers (mean scores < 2).

### 4.4 Immediate impact of PPI from the user representatives’ perspective

In the focus group, the Refugee Advisors conveyed a perceived impact on the research development process. They repeatedly spoke of how their input could be ‘useful’ to the research team. They described being helpful and expressed how their presence and their contributions, which they often termed as ‘information’, were advantageous to the research meeting. Their purpose within the meeting seemed to be clear; they were present to give their personal perspective on the different aspects of the research design.

I gave them information that could be **useful.** (RA1, refugee mother)

They articulated the importance of sharing their experiences, both for the research process and for them personally. They attributed great value to their ‘stories’ and the details of their life‐experiences in their home countries, during their flight to Sweden, and since arriving in Sweden. They expressed a desire for others to know, and to learn from, their experiential knowledge.

I feel that my life is like a tale. A tale I want others to know. (RA2, refugee youth)

However, wider involvement was proposed, suggesting a perceived limitation to their input. In other words, they can only express their own experiences and cannot speak for all refugees.

You could invite more families. More families can bring more opinions. The more opinions you get, the more useful information you will receive. (RA3, refugee father)

Two primary themes emerged from the focus group when considering the impact on the advisors. First, they indicated perceived equality and acceptance. Their comments inferred that they were received as valid members of the research group, and there was a sense of equal status across everyone who participated in the research meeting.

There was a very welcoming atmosphere, welcoming towards everyone. (RA3, refugee father)

Second, they spoke as though they had acquired knowledge from the process. They spoke directly about ‘knowledge’ and ‘information’ and benefitting from the research discussion.

I benefited myself from their questions. (RA1, refugee mother)

There was a strong underlying sense of appreciation, which was inferred as related to both the perceived equality and acceptance and the acquired knowledge. They gave anecdotal accounts of how the research team had been kind throughout the day and their gratitude for this. They also expressed a sense of comfort in the setting, which was taken to represent enjoyment in being part of the research meeting and overall process.

I felt like an honorary guest…Even if there will be one more hour, I am staying here happily [laughs]. (RA4, refugee mother)

There was also a sense of empowerment, clearly conveyed. The advisors spoke as though they felt accepted and valued by the researchers, which in turn made them feel stronger and more confident.

First, I am illiterate, I cannot read and write and here I got the chance to be among educated people that are so knowledgeable in their field and that made me feel like a human being. There cannot be a better feeling when you are sitting with educated people, and before you were feeling like nothing and now you feel as a human […] There is a bad feeling, for example my teacher of social sciences at school, just because she knows that I am illiterate, she ignores me when I ask questions to her. She answers to everybody except me. So I do not feel like I am an effective person […] I felt that today. (RA3, refugee father)

A particular outcome that was interpreted as a manifestation of empowerment was the intention for social sharing and how this indicated a desire for social change. All of the advisors spoke about how they would tell others of their experience participating in the research meeting. They expressed how they would share both the information they felt they gained from the day, and the positive feelings that came from taking part. It seemed the intention of this social sharing was for other refugee families to learn and to share the positivity that had arose from the meeting.

I will also spread the knowledge and the information to my course mates at school and tell them about the positive experiences we had. (RA2, refugee youth)

## 5 DISCUSSION

### 5.1 Reflections on case study results

Findings were largely positive, with notably high levels of positive interactions and invitations to participate and low levels of ideas being ignored/treated with disregard and decisions made without the input of advisors. There were clear examples of how the advisors influenced research development. The advisors described a perceived impact on the research, equality and acceptance, and knowledge gain. A sense of appreciation and empowerment was also interpreted.

#### 5.1.1 Interpersonal relations

There were high levels of positive interactions. However, challenges in interpersonal relations were seen across (a) references to advisors expertise, (b) linguistic barriers to participation and (c) advisors showing a lack of interest/being disengaged. First, it seems researchers overestimated how frequently they referred to the expertise of the advisors. Looking across the other observation protocol categories (*Nature of advisor contributions* and *How advisors guided research development*), we can see that researchers also appeared to overestimate the number of ‘irrelevant contributions from the advisors’ and underestimate how often they incorporated advisors’ ideas into research planning. Taken together, these results could be interpreted as perceived ‘ability’ and/or ‘control’ by the researchers. When evaluating researchers’ role in knowledge co‐production, Pohl et al^26^ identified two key challenges of ‘power’ and ‘integration’. Researchers have to assume new roles, quite apart from their traditional roles as academic authorities, as reflective scientist, intermediary and facilitator. The present findings allude to these challenges.

‘Linguistic barriers to participation’ were commonly observed, which included issues arising from interpretation. The interpreter used in the present study had professional knowledge of the research subject. This was advantageous in that he was clearly passionate about the topic and establishing a good level of trust with the Refugee Advisors, which Vara and Patel^3^ describe as important in order to facilitate frank discussion when using an interpreter in a research context. However, at times the passion led to the interpreter overstepping his role and offering personal insights. Investment in developing a close link with the interpreter is essential to the research process.^3^ Other interpreter‐related challenges to bear in mind are (a) the slowing effect on the pace of conversation and (b) the physical positioning in the room required for the advisors to be able to interact with the interpreter. The latter could affect the power balance of the meeting as physical grouping could lead to a subconscious ‘them and us’ mentality.

The majority of the observed ‘lack of engagement’ behaviours were displayed by the youth advisor, which could be a result of being in an adult‐centred environment and the prolonged format of the full‐day meeting.

#### 5.1.2 Nature of advisor contributions

There were high levels of 'invitations to speak' and 'taking the initiative to speak' and low levels of 'passively agreeing with researchers'. However, the questionnaire, and to some extent the observation, results indicated the relevance of input from Refugee Advisors was lacking at times. There are a couple of potential reasons for this. First, it could be connected to cultural understanding of ‘mental health’. If refugees have not engaged with mental health services in a Western context, they may not be familiar with the psychological language that is often used. Another issue could be the Refugee Advisors’ understanding of their role and the involvement process. This could be developed with specific PPI training. Much of the current training for patients and the public focuses on addressing the gaps in their knowledge and awareness about how research works and how public involvement adds value; however, there is a need to identify and develop the ‘soft skills’ required to influence researchers effectively.^27^ Researchers often don't know what they don't know, which makes it challenging to identify ahead of time which aspects of the patient's/ member of the public's lived experience should be shared.^27^ Effective involvement, and a consensus on the relevance of input, is underpinned by the patient/ member of the public being skilled in identifying what the researcher doesn't know or has assumed.

#### 5.1.3 How advisors guided research development

There were clear examples of how the advisors influenced research development. The form and nature of these contributions, arising from the observational notes, can be beneficial to future PPI conduct and research efforts. First, the examples (i.e. recruitment strategy, cultural brokerage among study group participants, outcome measure validation) can serve as inspiration to other research teams with regard to the type of advice PPI representatives can provide. Second, they can provide the grounding for a longitudinal evaluation of PPI. For instance, do the recruitment strategies lead to an increased number of participants recruited? Or, do the cultural brokerage strategies improve group cohesion and, ultimately, improve participant retention?

#### 5.1.4 Immediate impact of PPI from the user representatives’ perspective

The advisors described a perceived impact on the research, equality and acceptance, and knowledge gain. These positive perceptions are indicators of good quality involvement that are not always seen in descriptions of PPI activity; for instance, there have been reports of assumed lack of knowledge and associated frustration from user representatives.^19^, ^20^, ^21^, ^22^ The appreciation and empowerment arising from the active involvement described aligns with previous research, in which personal benefits to user representatives including feeling empowered are reported.^28^, ^29^, ^30^, ^31^, ^32^, ^33^

#### 5.1.5 Case study methods

When considering the application of the multi‐method approach, there are some notable limitations. First, the placement of the focus group at the end of the 8‐hour meeting may have resulted in a relatively brief discussion (35 minutes) as the Refugee Advisors were likely tired. The reason for this format being chosen was twofold; (a) to capture the immediate insights of the Refugee Advisors and (b) to avoid the inconvenience of them having to travel on a separate occasion. Second, only the researchers completed the Active Involvement of Users in Research Questionnaire. It would have been insightful to compare the questionnaire responses from the researchers and the Refugee Advisors; however, we were conscious of the burden placed on the Refugee Advisors, given the additional time required to conduct the focus group, and the questionnaire was not available in Arabic at the time of the research meeting.

## 6 APPRAISAL OF THE ACTIVE INVOLVEMENT OF USERS IN RESEARCH OBSERVATION SCHEDULE AND QUESTIONNAIRE

The observation protocol and questionnaire are promising tools for evaluating group dynamic characteristics of PPI. The present study was a pilot and will inform further development. The observer in the present study was unable to code all observations live and thus reviewed the protocol after the meeting to code remaining observations. Although this is often reported in observation studies, refinement of the coding guidance and pro‐forma layout could improve usability. A scoring system, as commonly utilized in observational protocols, is also required. The inter‐rater reliability of the observation protocol should be evaluated. The validity of corresponding questionnaire items measuring perception of PPI group dynamic characteristics by meeting participants should be tested, and how far these correlate with observations should be further explored. Comparison of the observational and questionnaire data from the present study indicates reasonable concurrent validity. However, focusing solely on the *frequency* of the characteristics and assuming equal weighting across them may not be appropriate. The significance of how advisors guide research development is conceivably greater than interpersonal relations or the nature of advisor contributions, given that the purpose of PPI is to inform research design and conduct. Therefore, the weighting of the observation protocol scoring system requires careful consideration. The practicality and validity of using the questionnaire with *all* individuals involved in the PPI process should be explored. This further triangulation of data could help to understand differences in perceptions and consensual variation across items, which will inform score weighting. Wider scale application (with more advisors in further meetings) will inform the development process and improve generalizability of the tools. Moreover, the application with other communities in other contexts will be important.

## 7 CONCLUSIONS AND IMPLICATIONS OF THE RESEARCH

Case study findings indicate a need for thorough PPI preparation, perhaps skill‐based training,^27^ for everyone involved in the PPI process. The case study focused on PPI in the research development phase. To gain a full understanding of the impact on the research process, a longer‐term evaluation is required. Prospective longitudinal studies could capture how impact changes over time. This relates to impact on the patients or members of the public *and* on the research project (e.g. recruitment rates, participant‐reported acceptability). The advisors in the present study indicated high levels of appreciation and empowerment; however, this impact could diminish over time and the ‘feedback loop’ in understanding how their involvement has actually affected the research could be an important part of the process in maintaining both appreciation and empowerment.

The methodological approach piloted in this study offers a promising way to not only evaluate the involvement of refugees in the development of mental health research, but PPI more broadly. As described above, the research tools require further refinement and validation. This would be greatly informed by a renewed review of the literature. The reviews currently available on evaluative PPI literature include research published up to 2012.^8^, ^9^ Given the increased interest in PPI over the last few years, with many research funders making PPI mandatory and the inception of PPI special issues and dedicated journals, it is reasonable to assume the knowledge base has grown.

## CONFLICT OF INTEREST

The authors declare that they have no conflicts of interest.

## Data Availability

The data that support the findings of this study are available from the corresponding author upon reasonable request.
